# Assessment of Longevity and Lifetime Productivity of Local Cattle Breeds in Relation to International Breeds

**DOI:** 10.3390/ani15223312

**Published:** 2025-11-17

**Authors:** Wioletta Sawicka-Zugaj, Witold Chabuz, Joanna Barłowska, Sebastian Mucha, Andrzej Bochniak

**Affiliations:** 1 Department of Cattle Breeding and Genetic Resources Conservation, University of Life Sciences in Lublin, 20-950 Lublin, Poland; wioletta.sawicka@up.edu.pl; 2 Department of Quality Assessment and Processing of Animal Products, University of Life Sciences in Lublin, 20-950 Lublin, Poland; joanna.barlowska@up.edu.pl; 3 Polish Federation of Cattle Breeders and Dairy Farmers in Warsaw, 00-515 Warsaw, Poland; s.mucha@pfhb.pl; 4 Department of Applied Mathematics and Computer Science, University of Life Sciences in Lublin, 20-950 Lublin, Poland; andrzej.bochniak@up.edu.pl

**Keywords:** local breeds, production properties, longevity

## Abstract

This study was based on data obtained from a total of 9518 cows belonging to seven cattle breeds: local breeds—Polish White-Backed (249), Polish Red (269), Polish Black-and-White (255), and Polish Red-and-White (290)—and international breeds—Polish Holstein-Friesian (5917), Jersey (940), and Simmental (1598). The breeds were characterised in terms of the following parameters: length of life, length of productive life, milking life, longevity index, percentage share of yield in the first 305-day lactation and first complete lactation in the lifetime yield, and functional longevity. Reasons for culling and the relationship between the length of life/productive life and milk performance parameters were determined as well. The analysis of the length of life and length of productive life in seven different cattle breeds kept in Poland unequivocally demonstrates that local breeds clearly stand out in this regard in comparison to international breeds. They may in the future constitute a valuable gene reservoir for improving longevity in other breeds. The length of life of Polish White-Backed and Polish Red cows was 2817 days and 3607 days, respectively, while that of Polish Holstein-Friesian and Jersey cows was only 2131 and 1956 days, respectively, and the most common cause of culling of cows in Poland (39.07%), irrespective of breed, was reproductive problems. The favourable results of parameters related to the longevity of the local breeds of Polish Red and White-Backed show that they can become a tool for improving the longevity of international breeds.

## Simple Summary

Longevity is one of the most important functional features, both from an economic and production perspective. Although cattle can live to be around 20 years old under natural conditions, the high intensity of production has significantly shortened this period. The aim of this study was to analyse parameters associated with the length of life and productivity of cows, taking into account the breed factor. The analyses carried out allowed us to conclude that native breeds clearly differ from high-production breeds, achieving better parameters regarding longevity, length of life in the herd, and the share of the first lactation in the entire productive life of the animal. The demonstrated tendency of native breeds towards longevity is an additional advantage to those already known, associated with the production of high-quality material.

## 1. Introduction

Longevity is one of the most important functional traits of dairy cows. According to Groen et al. [1] and Boettcher [2], functional traits are phenotypic traits in animals that increase the profitability of production while reducing its costs. However, due to the fact that the intensive development of agriculture has also involved significant intensification of dairy cattle farming and continually increasing milk yield, functional traits were a lower priority for some time [2,3]. Currently, however, two trends guiding global milk production can be observed. On the one hand, the goal of producers, understandably, is to produce as much milk as possible, while on the other hand, informed consumers are looking for a product that is not only safe but of high quality and beneficial for their health and well-being [4,5]. On the assumption that healthy food can only be obtained from healthy animals, the dairy sector has begun to focus on animal health and welfare. Therefore, greater attention has started to be focused on functional traits such as longevity, fertility, and health, and subsequently, selection indices have been updated [6,7].

Longevity in dairy cows refers to the length of the period from the first calving to the cow’s removal from the herd, which, according to Najafabadi et al. [8], is most often due to unsatisfactory yield. Although the natural length of life of cows can even exceed 20 years, highly productive cows, e.g., Holstein-Friesians, are only used 2–3 years after reaching productive age [9]. An alternative to improving the longevity of high-production dairy cows is crossbreeding with breeds characterised by a longer life and production period. Most of these studies were conducted on the Holstein breed [10,11,12,13,14,15,16], and the obtained results showed that the F1 generation was characterised by more favourable longevity parameters.

According to analyses by Horn et al. [17] in Austrian Simmentals, cattle reach their maximum yearly production in their fifth lactation, and the highest economic outcomes, taking into account longevity and milk yield, are not reached until the sixth lactation. Hence, from an economic perspective, longevity is an extremely important trait for the cattle farmer and, in some cases, such as in Simmental cattle in Austria, even the most important [18]. According to Strapák et al. [19], high longevity of Bavarian Simmental reduces farming costs and helps to maximise the animal’s milk production at the most appropriate age. An extensive analysis of longevity traits carried out by Hu et al. [20] showed that scientists have been studying the problem of longevity in cattle for many years, introducing several definitions and methods for its assessment, which have been divided into two groups: those pertaining to the health and length of life of animals and those associated with stability in the herd. The former includes the length of life in the herd, i.e., from birth to culling [21,22]; length of productive life—from first calving to slaughter [23]; and the number of days in milk in the cow’s entire milking life, excluding all dry periods [24]. Stability traits include remaining in the herd up to a certain age after the first calving [25] or the cow’s ability to give birth at least three times by the age of 76 months [26]. However, the length of the productive life of cows and the time they remain in the herd depend on many factors, including those associated with the animals’ use, i.e., lactation yield, health, conformation traits, and reproductive performance, as well as environmental factors, such as diet, management, the agricultural policies of a given country, or the price of milk [27,28,29].

The aim of this study was to analyse parameters associated with the length of life and productivity of cows, taking into account the breed factor.

## 2. Materials and Methods

### 2.1. Material and Animal Selection Criteria

The study material consisted of data pertaining to the production parameters and length of productive life of 9518 cows belonging to seven cattle breeds, Polish White-Backed (BG); Polish Red (RP); Polish Black and White (ZB); Polish Red and White (ZR); Polish Holstein-Friesian (HO); Simmental (SM); and Jersey (JE), kept on 243 farms throughout Poland. Native breeds were kept on small, extensive farms with 4–20 cows, while international breeds were kept on large intensive dairy farms, keeping on average 80 cows for HO, 50 for JE, and 40 for SM. Only one breed was kept on each of the analysed farms. The cows selected for analysis were sired by a total of 2209 bulls (Table 1). The data used for the analysis were obtained from the ITC system of the Polish Federation of Cattle Breeders and Dairy Farmers—FedInfo. They included information on the animals’ dates of birth and culling, the reason for culling, the date of each calving, lactation yield, and the chemical composition of the milk.

The following criteria were applied to select animals for further analysis:

– Animals used in 2004–2024 were considered;
– In the case of local breeds (BG, RP, ZB, and ZR), the entire population was analysed, and farms with at least 5 cows registered in breed books were chosen;
– In the case of HO, 100 farms with at least 50 cows registered in the breed book were randomly selected;
– In the case of JE, all farms with at least 10 cows registered in the breed book were selected;
– In the case of SM, 30 farms with at least 30 cows registered in the breed book were randomly selected;
– Documented lineage going back at least two generations was available;
– Information from birth to culling was available, i.e., date of birth, calving dates, dry period dates, and the date and cause of culling;
– Information on at least one 305-day lactation was available, i.e., milk yield and yield and content of protein, fat, and lactose.

### 2.2. Calculation of Longevity and Productivity Parameters

Once the data were obtained and organised, they were used to determine the following longevity parameters:

– Length of life, as the period from birth to culling (days);
– Productive life, as the period from first calving to culling (days);
– Milking life, as the period from first calving to culling, excluding dry periods (days);
– Longevity index (LI), according to the following formula:

$$LI=\frac{ML}{LT}\times 100\%$$

where

ML—number of days in milk in the cow’s life;

LT—length of cow’s life.

– Percentage share of the yield in the first 305-day lactation in lifetime yield (L1), according to the following formula:

$$L1=\frac{MY305}{LY}\times 100\%$$

where

MY305—milk yield in the first 305-day lactation (kg);

LY—lifetime milk yield (kg).

– Percentage share of the yield in the first complete lactation in lifetime yield (L2), according to the following formula:

$$L2=\frac{1FL}{LY}\times 100\%$$

where

1FL—yield in first complete lactation (kg);

LY—lifetime milk yield (kg).

– Functional longevity (FL), according to the following formula:

$$FL=\frac{L1D}{UD}\times 100\%$$

where

L1D—length of lactation (days);

UD—number of days of productive life.

The following were determined as well:

– Reason for culling in each breed;
– Milk performance parameters in relation to lifetime production: milk yield (kg); yield (kg) and content (%) of protein; yield (kg) and content (%) of fat; yield (kg) and content (%) of lactose; yield (kg) and content (%) of dry matter;
– Milk performance parameters per day of life: milk yield (kg), protein yield (kg), fat yield (kg), lactose yield (kg), dry matter yield (kg);
– Milk performance parameters per day of productive life: milk yield (kg), protein yield (kg), fat yield (kg), lactose yield (kg), dry matter yield (kg).

### 2.3. Statistical Analysis

The data obtained from the reproductive and dairy performance evaluations of cows belonging to each breed were systematically compiled into a comprehensive database for statistical analysis. All statistical computations were performed using the STATISTICA version 13.3 (StatSoft Inc., Tulsa, OK, United States) Before performing, data were tested for compliance with the assumptions of normality and homogeneity of variances, confirming that the distribution of data within groups was approximately normal.

To compare the mean values of each reproductive parameter across the different breeds, a one-way analysis of variance (ANOVA) was employed. The analysis was conducted using two approaches. One analysis considered the division of breeds into two groups: local breeds and international breeds as an independent factor. The second, more detailed approach considered individual breeds as an independent factor. This approach allowed for the detection of statistically significant differences in performance traits attributable to breed effects.

To determine pairwise differences between breed means, Tukey’s post hoc test for unequal groups was applied. Mean values in tables denoted by different superscript letters (e.g., a, b, c) were interpreted as significantly different according to Tukey’s multiple comparison procedure.

To examine the strength and direction of linear relationships between the analysed reproductive and productive parameters and the breed factor, Pearson’s correlation coefficients were calculated.

In all statistical tests performed, a significance level of 0.05 was assumed.

The results are presented as the mean ± standard deviation and standard error of mean.

## 3. Results

The most common reason for culling of cows in the herds analysed was reproductive problems, accounting for 39.07% on average among all breeds. As shown in Table 2, the percentage of culling for this reason ranged from 31.16% (HO) to 46.30% (RP) and 46.20% (ZR). The least common reason for removing cows from herds was their average milk yield, accounting on average for 3.40%; the percentage of cows culled for this reason was highest in RP (5.56%) and ZB (5.10%), while no such cases were noted for ZR. Culling due to metabolic diseases (6.30%) and old age (6.50%) was also relatively rare, with the highest culling rates due to metabolic problems recorded in JE (20.37%) and old age in ZR (14.56%). Old age was the least likely factor to be considered for culling in HO (2.79%) and JE (2.78%) breeds. Udder diseases proved to be a significant reason for culling in HO, ZB, and JE: 30.69%, 25.51%, and 24.54%, respectively. A considerable number of cows culled due to limb disorders (17.05%) and accidents (12.90%) were noted in SM.

As can be seen in Table 3, local breeds were characterised by significantly higher indicators of length of life, length of productive life, and length of milking life (2981, 2110, and 1823 days, respectively) than international breeds (2160, 1321, and 1309 days, respectively). These differences are statistically significant (*p* < 0.05).

The average length of life in the seven breeds was 2223 days, i.e., just over six years, with particularly high values for RP (3607 days) and ZR (3165 days) and the lowest in JE (1956 days) (Table 4). The average length of productive life in the group was 1381 days; the international breeds JE and HO were used for the shortest time (1184 and 1295 days, respectively), while the values were intermediate for SM (1569 days), ZB (1626 days), and BG (1960 days) and highest for ZR and RP (2295 and 2773 days, respectively). Analysis of the milking period also showed the lowest values for HO and JE, i.e., 1281 and 1200 days, respectively, and the highest for the local breed RP—2384 days.

Analysis of lifetime milk performance traits revealed that international breeds had higher milk yields (28,174.57 kg), fat yields (1131.53 kg), protein yields (963.33 kg), and lactose yields (1344.75 kg) compared to local breeds (Table 5). The results were similar for the individual chemical components, with the exception of fat, which was higher in local breeds (4.18%). These differences are statistically significant (*p* < 0.05).

Analysis of lifetime milk performance traits revealed significant differences in means (*p* < 0.05) between all cattle breeds analysed (Table 6). The lifetime amount of milk produced ranged from 21,439.55 kg in ZB to 29,617.77 kg for HO. Besides HO, very good lifetime yield results were noted for ZR (25,586.80 kg), BG (25,595.89), and SM (25,973.18). JE cows had the statistically significant (*p* < 0.05) highest lifetime average dry matter content in the milk (14.60%), including the highest content of protein (3.9%) and fat (5.21%). The average dry matter content in the milk was lowest in ZR (12.60%), including protein content (3.23%), and in HO (12.70%), including fat content (3.89%).

The values of all milk performance parameters per day of life in international breeds (HO, SM, and JE) were significantly (*p* < 0.05) different from the values obtained for local breeds (BG, RP, ZB, and ZR) (Table 7). In all analysed parameters, i.e., milk yield and the yield and content of basic chemical components, higher values were recorded for international breeds.

The results for milk performance parameters per day of life of cows in all analysed breeds are presented in Table 8. HO cows had the best results for daily lifetime milk production, except for fat yield, which was highest in JE (0.53 kg). It should be noted that HO cows produced more than 50% more milk than RP cows (13.23 vs. 6.47 kg), which had the lowest values among all the breeds for all traits analysed. Analysis of milk performance traits per day of life revealed significant differences in means (*p* < 0.05) between all cattle breeds analysed.

Similarly to the results for milk performance parameters per day of life, international breeds were characterised by higher production indices, i.e., milk yield (21.88 kg), fat (0.87 kg), protein (0.75 kg), lactose (1.05 kg), and dry matter (2.83 kg), as well as milk yield per day in milking life (24.27 kg) compared to local breeds (Table 9). The differences between local and international breeds were statistically significant (*p* < 0.05).

Analysing the results of individual breeds, it was found that the lowest values for these parameters were obtained for RP, i.e., milk—8.84 kg, fat—0.38 kg, protein—0.29 kg, lactose—0.41 kg, and dry matter—1.13 kg, as in the case of day of milking life (11.20 kg of milk) (Table 10). The highest values, except for fat (JE—0.95 kg), were shown for HO: for yield of milk (23.39 kg), protein (0.79 kg), lactose (1.12 kg), and dry matter (2.97 kg) per day of productive life and milk yield per day of milking life (27.05 kg) (in *p* < 0.05).

Table 11 and Table 12 present the results for functional longevity (FL), the share of the first 305-day lactation yield in the lifetime yield (L1), the share of the first complete lactation yield in the lifetime yield (L2), and the longevity index (LI) for all breeds analysed. In the case of functional longevity and the longevity index, the best results can be seen for RP and BG. The length of the first lactation in these breeds amounted to 19.05% and 23.64% of their productive life, respectively, while the percentages were much greater in JE and HO, i.e., 47.72% and 43.48%. The RP breed also had the highest percentage of days in milk relative to length of life (61.18%), which was statistically significantly (*p* < 0.05) different from the results obtained for the other breeds, especially JE (52.86%). In the case of the proportion of milk yield in the first 305-day and first complete lactation in relation to lifetime yield, the highest, statistically significant (*p* < 0.05) results were obtained for JE: 41.38% and 50.70%, respectively. Their share was much lower in RP and BG: 18.98% and 22.19% for RP and 20.12% and 23.55% for BG.

The evaluation of longevity parameters was supplemented with the determination of correlations by Pearson’s method. Table 13 presents the correlations linking the number of days of productive life and length of life with lifetime milk performance traits. The correlations for yield of milk, fat, protein, lactose, and dry matter for all breeds analysed were very high (above 0.70) and statistically significant (*p* < 0.05). In the case of the percentage content of individual milk constituents, the correlations were low, but it is worth noting the correlation for lactose, which was negative in five breeds (BG, ZB, HO, SM, and JE). As the length of life and the length of productive life increased, the percentage content of lactose decreased. Analysis of the breed factor showed that, in the case of the yield of milk and its chemical constituents, the highest correlations are found for the Jersey breed (˃0.90).

The statistical correlations between milk yield and its chemical composition in the first complete lactation, and the length of productive life and life of cows are presented in Table 14. More than 87% of correlations were negative, and the lowest values in all breeds were noted for yield of lactose (from −0.13 in JE to −0.37 in ZR) and dry matter (from −0.15 in JE to −0.39 in ZR). This means that, as milk yield increases, a shorter life expectancy can be expected in cows.

## 4. Discussion

The issue of cattle longevity has been widely discussed in the scientific literature, particularly in reference to single-purpose breeds with high production potential [30,31,32]. Numerous studies show that the most common subject of research in various parts of the world is the Holstein-Friesian breed [24,32,33,34,35,36]. The present study, however, showed that it is the Jersey breed that has the shortest productive life, with even lower values shown in the United States (length of life—1528 days) [37] and Denmark (from 1010 to 1060 days of productive life) [38].

The changes that have taken place in dairy cattle farming in the last few decades, induced by numerous factors—higher demand for animal protein due to demographic growth [39], the resulting economic pressure, and, finally, enormous technological advances—have led to significant changes in how long animals are kept in herds [38,40,41]. This time period is much shorter than the natural life expectancy of cattle (about 20 years) [29,42]. According to the world literature [24,43,44], the length of life of cows has drastically decreased in countries with highly intensive milk production. In the present study, the analysis of the length of life and length of productive life of seven cattle breeds kept in Poland unequivocally demonstrates that local breeds clearly stand out in this regard in comparison to international breeds. This may be a direct consequence of their adaptation to the prevailing climate conditions where they live, as pointed out by Biscarini et al. [45], Strandén et al. [46], and Żmija [47]. This is particularly true of Polish Red, which, like the German Red and Czech Red breeds, is one of the oldest local cattle breeds in Central Europe [48]. The long productive life of red cattle (2966.2 days) has also been described by Cielava et al. [49] in their analysis of the lifetime productivity and longevity of breeds kept in Latvia.

The length of life of cows in a herd and, thus, the length of their productive life depend on farmers’ decisions based on an assessment of factors such as production level, health, reproductive potential, and the availability of replacement animals [29,50]. Reasons for culling have been analysed in many countries [51,52,53], especially with respect to farm economics [54,55,56]. The present study showed that the most common reason for culling of cows in Poland, irrespective of breed, was reproductive problems. According to Armengol and Fraile [51], this group of disorders includes abortions, ovarian cysts, anovulation, and post-calving problems such as dystocia, uterine torsion, or uterine or vaginal prolapse. Many authors indicate that reproductive problems are a common reason for culling of dairy cows [51,52,57,58,59].

In highly productive breeds, the amount of milk produced in the first lactation very often determines how long the cow is kept in the herd [60,61]. The present study, however, showed a small negative correlation between milk yield in the first complete lactation and the length of the productive life and life of cows of all breeds analysed. Similar values were reported by Sawa et al. [62] (−0.02322). This may be indicative of the caution with which farmers in Poland approach culling of cows. According to Dhuyvetter et al. [63], hasty culling of cows prevents exploitation of their full production potential, which decreases the profitability of dairy cattle farming. It is worth noting, however, that the local breeds BG and RP had the most favourable functional longevity, reflecting the long duration of the first lactation in relation to the total productive life. This supports research by Litwińczuk et al. [64], which showed that local breeds do not achieve their highest milk yield until their third lactation. Moreover, according to Berry et al. [65], an analysis of the longevity of dairy cattle should take into account the high level of public interest in an ethical approach to milk production. Longevity is currently treated as an indicator of an animal’s welfare, providing information about the normal course of biological processes associated with its health [52].

Haworth et al. [66] report that cows producing large daily amounts of milk have much shorter lives than those producing less than 15 L, which clearly demonstrates the impact of an intensive production system on the length of life of animals in a herd. The present study showed that the length of productive life and length of life were positively correlated with lifetime milk yield and chemical composition in all breeds and that the HO and JE breeds, which produced the most milk per day of life, lived the shortest time. It is also important to take note of the differences in the number of days in milk between the HO and JE breeds and the RP breed in relation to their length of life. Bieber et al. [67] showed that cows of local breeds have a low metabolism during lactation, which is responsible for lower milk yield but leads to a longer productive life.

## 5. Conclusions

The native Polish Red and Polish White-Backed breeds are clearly distinguished from the highly productive breeds in terms of functional longevity, length of life in the herd, and share of the first lactation in the animal’s entire productive life. This conclusion can be drawn from current knowledge and the availability of tools for objective longevity estimation in breeds with varying production intensity. Although the profitability of production should combine the highest possible milk yield with longevity, we continue to observe an increase in lactation yield at the cost of an excessive burden on the body and, thus, a shorter life. Public interest in the welfare of farm animals and an increasing focus on the carbon footprint of animal production are prompting measures to reduce the introduction of new animals to herds to replace cows that have only completed one or two lactations. An alternative, especially in areas with specific or even harsh climate conditions, is local breeds. The demonstrated tendency of native breeds towards longevity is an additional advantage to those already known, associated with the production of high-quality material. Furthermore, combining the possibility of obtaining valuable raw materials with the use of local breeds to increase the longevity of high-yielding breeds can have a positive impact on their protection and conservation.

## Figures and Tables

**Table 1:** Characteristics of the analysed material.

Breeds	N Cows	N Farms	N Fathers
Local breeds	1063	104	109
BG	249	26	19
RP	269	32	66
ZB	255	27	9
ZR	290	19	15
International breeds	8455	139	2100
HO	5917	100	1692
JE	940	9	116
SM	1598	30	292
All	9518	243	2209

Note: BG—Polish White-Backed; RP—Polish Red; ZB—Polish Black and White; ZR—Polish Red and White; HO—Polish Holstein-Friesian; SM—Simmental; JE—Jersey.

**Table 2:** Distribution of reasons for culling of dairy cows in Poland due to death and slaughter in the years 2004–2024.

Reason for Culling	All (%)	BG (%)	RP (%)	ZB (%)	ZR (%)	HO (%)	SM (%)	JE (%)
Unknown	8.28	10.82	5.56	4.59	10.13	11.16	9.68	4.63
Infertility	39.07	37.11	46.30	41.35	46.20	31.16	40.55	32.87
Foot or leg problems	8.78	6.19	5.56	7.65	10.76	9.30	17.05	5.09
Metabolic diseases	6.30	3.09	4.63	5.10	1.9	5.58	1.84	20.37
Low productivity	3.40	3.09	5.56	1.53	0.00	4.21	3.23	5.09
Old age	6.50	6.19	12.50	5.61	14.56	2.79	3.23	2.78
Udder problems	19.25	21.13	10.65	25.51	9.49	30.69	11.52	24.54
Accidents	8.42	12.37	9.26	7.65	6.96	5.11	12.90	4.63

Note: BG—Polish White-Backed; RP—Polish Red; ZB—Polish Black and White; ZR—Polish Red and White; HO—Holstein-Friesian; SM—Simmental; JE—Jersey.

**Table 3:** Differences in length of life (days), length of productive life (days), and length of milking life (days) between local and international breeds kept in Poland.

	All	Local Breeds	International Breeds	Test Statistics
N	9518	1063	8455	F = 659.6
Length of life (days)	2223.24	2981.13 ^a^	2159.75 ^b^	*p* < 0.0001
	±823.14	±1152.90	±755.63	
	SEM = 8.4	SEM = 35.4	SEM = 8.2	
Length of productive life (days)	1381.50	2109.74 ^a^	1320.51 ^b^	F = 627.8
	±809.31	±1162.99	±740.65	*p* < 0.0001
	SEM = 8.3	SEM = 35.7	SEM = 8.1	
Length of milking life (days)	1400.37	1822.90 ^a^	1308.83 ^b^	F = 265.5
	±739.88	±954.94	±649.37	*p* < 0.0001
	SEM = 7.6	SEM = 29.3	SEM = 7.1	

Note: Different lowercase letters in the same row indicate statistical differences between breeds (*p* < 0.05). SEM—standard error of mean.

**Table 4:** Breed differences in length of life (days), length of productive life (days), and length of milking life (days) in Poland.

Breed	BG	RP	ZB	ZR	HO	SM	JE	Test Statistics
N	249	269	255	290	5917	1598	940	
Length of life (days)	2817.2 ^d^	3606.6 ^f^	2545.1 ^c^	3165.0 ^e^	2131.2 ^b^	2478.9 ^c^	1955.9 ^a^	F = 126
	±914.8	±1336.6	±883.7	±1284.2	±722.2	±869.0	±701.9	*p* < 0.0001
	SEM = 58.0	SEM = 81.5	SEM = 55.3	SEM = 75.4	SEM = 9.4	SEM = 21.7	SEM = 22.9	
Length of productive life (days)	1960.2 ^d^	2773.0 ^f^	1625.5 ^c^	2295.0 ^e^	1294.7 ^b^	1568.9 ^c^	1184.4 ^a^	F = 113.9
	±920.9	±1334.3	±858.3	±1312.0	±709.0	±870.6	±699.9	*p* < 0.0001
	SEM = 58.4	SEM = 81.4	SEM = 53.8	SEM = 77	SEM = 9.2	SEM = 21.8	SEM = 22.8	
Length of milking life (days)	1626.3 ^b^	2383.7 ^d^	1511.4 ^b^	1944.9 ^c^	1280.6 ^a^	1514.2 ^b^	1200.0 ^a^	F = 84.1
	±784.80	±1058.7	±719.71	±1104.1	±612.7	±762.6	±589.3	*p* < 0.0001
	SEM = 49.7	SEM = 64.5	SEM = 45	SEM = 64.8	SEM = 8	SEM = 19.1	SEM = 19.2	

Note: BG—Polish White-Backed; RP—Polish Red; ZB—Polish Black and White; ZR—Polish Red and White; HO—Holstein-Friesian; SM—Simmental; JE—Jersey. Different lowercase letters in the same row indicate statistical differences between breeds (*p* < 0.05). SEM—standard error of mean.

**Table 5:** Differences in milk, fat, protein, lactose, and dry matter yield (kg) and fat, protein, lactose, and dry matter content (%) between local and international breeds kept in Poland.

	All	Local Breeds	International Breeds	Test Statistics
Milk yield (kg)	27,865.81	24,179.60 ^a^	28,174.57 ^b^	F = 36.5
	±16,446.62	±13,266.15	±16,649.21	*p* < 0.0001
	SEM = 168.6	SEM = 406.9	SEM = 181.1	
Fat (kg)	1121.12	996.92 ^a^	1131.53 ^b^	F = 25.9
	±657.66	±571.39	±663.36	*p* < 0.0001
	SEM = 6.7	SEM = 17.5	SEM = 7.2	
Fat (%)	4.08	4.18 ^a^	4.07 ^b^	F = 15.1
	±0.68	±0.49	±0.69	*p* = 0.0001
	SEM = 0.007	SEM = 0.015	SEM = 0.008	
Protein (kg)	949.96	790.32 ^a^	963.33 ^b^	F = 60.2
	±555.15	±450.53	±561.00	*p* < 0.0001
	SEM = 5.7	SEM = 13.8	SEM = 6.1	
Protein (%)	3.42	3.30 ^a^	3.43 ^b^	F = 123.7
	±0.29	±0.26	±0.29	*p* < 0.0001
	SEM = 0.003	SEM = 0.008	SEM = 0.003	
Lactose (kg)	1326.11	1103.51 ^a^	1344.75 ^b^	F = 59.3
	±779.46	±613.28	±788.99	*p* < 0.0001
	SEM = 8.0	SEM = 18.8	SEM = 8.6	
Lactose (%)	4.76	4.68 ^a^	4.77 ^b^	F = 139.3
	±0.19	±0.30	±0.17	*p* < 0.0001
	SEM = 0.002	SEM = 0.009	SEM = 0.002	
Dry matter (kg)	3586.23	3026.89 ^a^	3633.08 ^b^	F = 52.8
	±2076.19	±1680.82	±2099.27	*p* < 0.0001
	SEM = 21.3	SEM = 51.6	SEM = 22.8	
Dry matter (%)	12.95	12.84 ^a^	12.96 ^b^	F = 10.2
	±0.95	±0.91	±0.96	*p* = 0.0014
	SEM = 0.01	SEM = 0.028	SEM = 0.01	

Note: Different lowercase letters in the same row indicate statistical differences between breeds (*p* < 0.05). SEM—standard error of mean.

**Table 6:** Breed differences in milk, fat, protein, lactose, and dry matter yield (kg) and fat, protein, lactose, and dry matter content (%) in Poland.

Trait	BG	RP	ZB	ZR	HO	SM	JE	Test Statistics
Milk yield (kg)	25,595.89 ^abe^ ±12,717.09	24,716.50 ^abc^ ±13,528.94	21,439.55 ^ab^ ±11,697.09	25,586.80 ^abd^ ±15,969.83	29,617.77 ^cde^ ±17,185.71	25,973.18 ^b^ ±14,649.66	21,761.70 ^a^ ±13,372.09	F = 43.4 *p* < 0.0001
	SEM = 805.9	SEM = 824.9	SEM = 732.5	SEM = 937.8	SEM = 223.4	SEM = 366.5	SEM = 436.1	
Fat (kg)	1002.17 ^ab^ ±570.55	1067.92 ^ad^ ±592.83	919.25 ^a^ ±506.79	1021.35 ^ac^ ±642.32	1146.25 ^bcd^ ±669.31	1054.76 ^a^ ±593.36	1131.18 ^bcd^ ±698.76	F = 8.2 *p* < 0.0001
	SEM = 36.2	SEM = 36.1	SEM = 31.7	SEM = 37.7	SEM = 8.7	SEM = 14.8	SEM = 22.8	
Fat (%)	4.10 ^bc^ ±0.44	4.26 ^cd^ ±0.62	4.28 ^d^ ±0.42	4.01 ^ab^ ±0.37	3.89 ^a^ ±0.57	4.07 ^b^ ±0.40	5.21 ^e^ ±0.55	F = 812.0 *p* < 0.0001
	SEM = 0.028	SEM = 0.038	SEM = 0.026	SEM = 0.022	SEM = 0.007	SEM = 0.01	SEM = 0.018	
Protein (kg)	817.35 ^ab^ ±466.33	822.83 ^acd^ ±446.30	714.35 ^a^ ±389.48	827.06 ^acd^ ±517.50	995.29 ^d^ ±575.34	885.92 ^bc^ ±492.45	855.69 ^a^ ±523.93	F = 24.0 *p* < 0.0001
	SEM = 29.6	SEM = 27.2	SEM = 24.4	SEM = 30.4	SEM = 7.5	SEM = 12.3	SEM = 17.1	
Protein (%)	3.32 ^ab^ ±0.19	3.28 ^ab^ ±0.39	3.32 ^ab^ ±0.20	3.23 ^a^ ±0.14	3.35 ^b^ ±0.22	3.42 ^c^ ±0.20	3.93 ^d^ ±0.24	F = 922.0 *p* < 0.0001
	SEM = 0.012	SEM = 0.024	SEM = 0.013	SEM = 0.008	SEM = 0.003	SEM = 0.005	SEM = 0.008	
Lactose (kg)	1144.65 ^a^ ±633.06	1123.83 ^ab^ ±597.30	1012.03 ^a^ ±545.69	1159.75 ^abc^ ±702.93	1413.90 ^c^ ±814.34	1242.85 ^b^ ±692.78	1033.19 ^a^ ±632.37	F = 48 *p* < 0.0001
	SEM = 40.1	SEM = 36.4	SEM = 34.2	SEM = 41.3	SEM = 10.6	SEM = 17.3	SEM = 20.6	
Lactose (%)	4.71 ^bc^ ±0.15	4.61 ^a^ ±0.54	4.72 ^bd^ ±0.15	4.66 ^ab^ ±0.17	4.76 ^d^ ±0.18	4.81 ^e^ ±0.14	4.75 ^cd^ ±0.14	F = 40.3 *p* < 0.0001
	SEM = 0.01	SEM = 0.033	SEM = 0.009	SEM = 0.01	SEM = 0.002	SEM = 0.004	SEM = 0.005	
Dry matter (kg)	3118.69 ^a^ ±1728.51	3124.24 ^ab^ ±1652.56	2793.55 ^a^ ±1518.77	3124.99 ^ab^ ±1889.71	3760.25 ^b^ ±2153.21	3354.65 ^a^ ±1860.30	3169.29 ^a^ ±1923.79	F = 24.6 *p* < 0.0001
	SEM = 109.5	SEM = 100.8	SEM = 95.1	SEM = 111	SEM = 28	SEM = 46.5	SEM = 62.7	
Dry matter (%)	12.82 ^ab^ ±0.57	12.83 ^ab^ ±1.54	12.99 ^b^ ±0.59	12.60 ^a^ ±0.50	12.70 ^a^ ±0.77	12.99 ^b^ ±0.56	14.60 ^c^ ±0.72	F = 879.2 *p* < 0.0001
	SEM = 0.036	SEM = 0.094	SEM = 0.037	SEM = 0.029	SEM = 0.01	SEM = 0.014	SEM = 0.023	

Note: BG—Polish White-Backed; RP—Polish Red; ZB—Polish Black and White; ZR—Polish Red and White; HO—Holstein-Friesian; SM—Simmental; JE—Jersey. Different lowercase letters in the same row indicate statistical differences between breeds (*p* < 0.05). SEM—standard error of mean.

**Table 7:** Differences in milk, fat, protein, lactose, and dry matter yield per day of life (kg) between local and international breeds kept in Poland.

	All	Local Breeds	International Breeds	Test Statistics
Milk yield/day of life (kg)	12.06	7.86 ^a^	12.41 ^b^	F = 594.2
	±4.77	±2.65	±4.74	*p* < 0.0001
	SEM = 0.049	SEM = 0.081	SEM = 0.052	
Fat yield/day of life (kg)	0.48	0.32 ^a^	0.50 ^b^	F = 619.4
	±0.18	±0.12	±0.18	*p* < 0.0001
	SEM = 0.002	SEM = 0.004	SEM = 0.002	
Protein yield/day of life (kg)	0.41	0.25 ^a^	0.43 ^b^	F = 746.7
	±0.16	±0.09	±0.16	*p* < 0.0001
	SEM = 0.002	SEM = 0.003	SEM = 0.002	
Lactose yield/day of life (kg)	0.58	0.36 ^a^	0.59 ^b^	F = 689.1
	±0.23	±0.13	±0.23	*p* < 0.0001
	SEM = 0.002	SEM = 0.004	SEM = 0.003	
Dry matter yield/day of life (kg)	1.55	0.98 ^a^	1.60 ^b^	F = 725.2
	±0.59	±0.35	±0.58	*p* < 0.0001
	SEM = 0.006	SEM = 0.011	SEM = 0.006	

Note: Different lowercase letters in the same row indicate statistical differences between breeds (*p* < 0.05). SEM—standard error of mean.

**Table 8:** Breed differences in milk, fat, protein, lactose, and dry matter yield per day of life (kg) in Poland.

Trait	BG	RP	ZB	ZR	HO	SM	JE	Test Statistics
Milk yield/day of life (kg)	8.96 ^bc^ ±2.74	6.47 ^a^ ±2.09	8.01 ^b^ ±2.53	7.43 ^ab^ ±3.02	13.23 ^e^ ±4.89	9.89 ^cd^ ±3.31	10.29 ^d^ ±3.33	F = 245.4 *p* < 0.0001
	SEM = 0.174	SEM = 0.127	SEM = 0.158	SEM = 0.177	SEM = 0.064	SEM = 0.083	SEM = 0.109	
Fat yield/day of life (kg)	0.34 ^b^ ±0.13	0.28 ^a^ ±0.09	0.34 ^b^ ±0.11	0.30 ^ab^ ±0.10	0.51 ^d^ ±0.18	0.40 ^c^ ±0.13	0.53 ^e^ ±0.17	F = 183.2 *p* < 0.0001
	SEM = 0.008	SEM = 0.005	SEM = 0.007	SEM = 0.006	SEM = 0.002	SEM = 0.003	SEM = 0.006	
Protein yield/day of life (kg)	0.28 ^b^ ±0.11	0.22 ^a^ ±0.07	0.27 ^b^ ±0.09	0.24 ^ab^ ±0.08	0.44 ^e^ ±0.16	0.34 ^c^ ±0.11	0.40 ^d^ ±0.13	F = 218.9 *p* < 0.0001
	SEM = 0.007	SEM = 0.004	SEM = 0.006	SEM = 0.005	SEM = 0.002	SEM = 0.003	SEM = 0.004	
Lactose yield/day of life (kg)	0.39 ^b^ ±0.15	0.30 ^a^ ±0.10	0.38 ^b^ ±0.12	0.34 ^ab^ ±0.11	0.63 ^d^ ±0.24	0.48 ^c^ ±0.16	0.49 ^c^ ±0.16	F = 259.5 *p* < 0.0001
	SEM = 0.01	SEM = 0.006	SEM = 0.008	SEM = 0.006	SEM = 0.003	SEM = 0.004	SEM = 0.005	
Dry matter yield/day of life (kg)	1.07 ^b^ ±0.41	0.83 ^a^ ±0.26	1.05 ^b^ ±0.33	0.92 ^ab^ ±0.30	1.68 ^e^ ±0.60	1.28 ^c^ ±0.42	1.50 ^d^ ±0.47	F = 220.7 *p* < 0.0001
	SEM = 0.026	SEM = 0.016	SEM = 0.021	SEM = 0.018	SEM = 0.008	SEM = 0.011	SEM = 0.015	

Note: BG—Polish White-Backed; RP—Polish Red; ZB—Polish Black and White; ZR—Polish Red and White; HO—Holstein-Friesian; SM—Simmental; JE—Jersey. Different lowercase letters in the same row indicate statistical differences between breeds (*p* < 0.05). SEM—standard error of mean.

**Table 9:** Differences in milk, fat, protein, lactose, and dry matter yield per day of productive life (kg) and milk yield per day in milking life (kg) between local and international breeds kept in Poland.

	All	Local Breeds	International Breeds	Test Statistics
Milk yield/day of productive life (kg)	21.12	12.00 ^a^	21.88 ^b^	F = 1399.7
	±7.04	±3.75	±6.70	*p* < 0.0001
	SEM = 0.072	SEM = 0.115	SEM = 0.073	
Fat yield/day of productive life (kg)	0.84	0.49 ^a^	0.87 ^b^	F = 1702.7
	±0.25	±0.16	±0.24	*p* < 0.0001
	SEM = 0.003	SEM = 0.005	SEM = 0.003	
Protein yield/day of productive life (kg)	0.72	0.39 ^a^	0.75 ^b^	F = 1741.2
	±0.24	±0.13	±0.22	*p* < 0.0001
	SEM = 0.002	SEM = 0.004	SEM = 0.002	
Lactose yield/day of productive life (kg)	1.01	0.55 ^a^	1.05 ^b^	F = 1498.1
	±0.35	±0.18	±0.33	*p* < 0.0001
	SEM = 0.004	SEM = 0.006	SEM = 0.004	
Dry matter yield/day of productive life (kg)	2.72	1.50 ^a^	2.83 ^b^	F = 1750.5
	±0.86	±0.49	±0.80	*p* < 0.0001
	SEM = 0.009	SEM = 0.015	SEM = 0.009	
Milk yield/day in milking life (kg)	22.58	14.79 ^a^	24.27 ^b^	F = 927.4
	±7.91	±7.07	±7.02	*p* < 0.0001
	SEM = 0.081	SEM = 0.217	SEM = 0.076	

Note: Different lowercase letters in the same column indicate statistical differences between breeds (*p* < 0.05).

**Table 10:** Breed differences in milk, fat, protein, lactose, and dry matter yield per day of productive life (kg) and milk yield per day in milking life (kg) in Poland.

Trait	BG	RP	ZB	ZR	HO	SM	JE	Test Statistics
Milk yield/day of productive life (kg)	13.61 ^c^ ±4.11	8.84 ^a^ ±2.38	13.37 ^bc^ ±3.04	11.05 ^ab^ ±2.28	23.39 ^f^ ±6.80	16.93 ^d^ ±4.28	18.36 ^e^ ±3.52	F = 529.5 *p* < 0.0001
	SEM = 0.26	SEM = 0.145	SEM = 0.19	SEM = 0.134	SEM = 0.088	SEM = 0.107	SEM = 0.115	
Fat yield/day of productive life (kg)	0.51 ^bc^ ±0.18	0.38 ^a^ ±0.11	0.57 ^c^ ±0.14	0.44 ^ab^ ±0.10	0.90 ^e^ ±0.24	0.69 ^d^ ±.17	0.95 ^f^ ±0.17	F = 501.3 *p* < 0.0001
	SEM = 0.011	SEM = 0.007	SEM = 0.009	SEM = 0.006	SEM = 0.003	SEM = 0.004	SEM = 0.006	
Protein yield/day of productive life (kg)	0.42 ^bc^ ±0.15	0.29 ^a^ ±0.08	0.45 ^c^ ±0.11	0.36 ^ab^ ±0.08	0.79 ^f^ ±0.23	0.58 ^d^ ±0.15	0.72 ^e^ ±0.14	F = 501.9 *p* < 0.0001
	SEM = 0.01	SEM = 0.005	SEM = 0.007	SEM = 0.005	SEM = 0.003	SEM = 0.004	SEM = 0.005	
Lactose yield/day of productive life (kg)	0.59 ^bc^ ±0.21	0.41 ^a^ ±0.11	0.63 ^c^ ±0.15	0.51 ^ab^ ±0.12	1.12 ^f^ ±0.34	0.82 ^d^ ±0.21	0.87 ^e^ ±0.17	F = 531.4 *p* < 0.0001
	SEM = 0.013	SEM = 0.007	SEM = 0.009	SEM = 0.007	SEM = 0.004	SEM = 0.005	SEM = 0.006	
Dry matter yield/day of productive life (kg)	1.61 ^bc^ ±0.55	1.13 ^a^ ±0.31	1.74 ^c^ ±0.40	1.38 ^ab^ ±0.32	2.97 ^f^ ±0.82	2.20 ^d^ ±0.55	2.68 ^e^ ±0.49	F = 520.8 *p* < 0.0001
	SEM = 0.035	SEM = 0.019	SEM = 0.025	SEM = 0.019	SEM = 0.011	SEM = 0.014	SEM = 0.016	
Milk yield/day in milking life (kg)	16.96 ^b^ ±6.61	11.20 ^a^ ±4.32	15.24 ^b^ ±3.42	14.86 ^b^ ±2.55	27.05 ^e^ ±7.37	20.03 ^c^ ±4.73	21.14 ^d^ ±4.03	F = 324.1 *p* < 0.0001
	SEM = 0.419	SEM = 0.263	SEM = 0.214	SEM = 0.737	SEM = 0.096	SEM = 0.118	SEM = 0.131	

Note: BG—Polish White-Backed; RP—Polish Red; ZB—Polish Black and White; ZR—Polish Red and White; HO—Holstein-Friesian; SM—Simmental; JE—Jersey. Different lowercase letters in the same column indicate statistical differences between breeds (*p* < 0.05). SEM—standard error of mean.

**Table 11:** Differences in functional longevity, share of the yield in the first 305-day lactation in lifetime yield, share of the yield in the first complete lactation in lifetime yield, and longevity index between local and international breeds kept in Poland.

	All	Local Breeds	International Breeds	Test Statistics
FL (%)	42.29	29.05 ^a^	43.15 ^b^	F = 109.6
	±26.82	±22.08	±26.87	*p* < 0.0001
	SEM = 0.275	SEM = 0.677	SEM = 0.292	
L1 (%)	35.89	24.80 ^a^	36.61 ^b^	F = 107.6
	±22.67	±18.17	±22.74	*p* < 0.0001
	SEM = 0.232	SEM = 0.557	SEM = 0.247	
L2 (%)	44.77	30.73 ^a^	45.67 ^b^	F = 108.9
	±28.54	±23.99	±28.57	*p* < 0.0001
	SEM = 0.293	SEM = 0.736	SEM = 0.311	
IL (%)	54.41	56.58 ^a^	53.94 ^b^	F = 28.5
	±11.22	±12.02	±10.98	*p* < 0.0001
	SEM = 0.115	SEM = 0.369	SEM = 0.119	

Note: FL—functional longevity; L1—share of the yield in the first 305-day lactation in lifetime yield; L2—share of the yield in the first complete lactation in lifetime yield; IL—longevity index (ratio of days in milk to length of life). Different lowercase letters in the same column indicate statistical differences between breeds (*p* < 0.05). SEM—standard error of mean.

**Table 12:** Breed differences in functional longevity, share of the yield in the first 305-day lactation in lifetime yield, share of the yield in the first complete lactation in lifetime yield, and longevity index between local and international breeds kept in Poland.

Parameter	BG	RP	ZB	ZR	HO	SM	JE	Test Statistics
FL (%)	23.64 ^a^ ±11.11	19.05 ^a^ ±17.00	38.07 ^bd^ ±26.18	32.38 ^abc^ ±25.46	43.48 ^cd^ ±26.51	37.34 ^b^ ±25.82	47.72 ^e^ ±29.28	F = 35.1 *p* < 0.0001
	SEM = 0.704	SEM = 1.037	SEM = 1.639	SEM = 1.495	SEM = 0.345	SEM = 0.646	SEM = 0.955	
L1 (%)	20.12 ^a^ ±9.52	18.98 ^a^ ±14.92	30.39 ^bc^ ±20.53	29.33 ^abcd^ ±24.57	36.71 ^b^ ±22.26	31.93 ^c^ ±21.84	41.38 ^d^ ±25.73	F = 33.7 *p* < 0.0001
	SEM = 0.603	SEM = 0.91	SEM = 1.286	SEM = 1.443	SEM = 0.289	SEM = 0.546	SEM = 0.839	
L2 (%)	23.55 ^a^ ±11.80	22.19 ^a^ ±19.61	39.91 ^bc^ ±27.85	35.30 ^abcd^ ±29.39	46.04 ^c^ ±28.15	39.22 ^b^ ±27.49	50.70 ^d^ ±31.17	F = 35.4 *p* < 0.0001
	SEM = 0.748	SEM = 1.196	SEM = 1.744	SEM = 1.726	SEM = 0.366	SEM = 0.688	SEM = 1.017	
IL (%)	55.41 ^ab^ ±11.74	61.18 ^c^ ±10.77	54.41 ^ab^ ±10.58	55.86 ^ab^ ±14.99	54.01 ^ab^ ±11.22	55.02 ^b^ ±11.83	52.86 ^a^ ±10.11	F = 12.6 *p* < 0.0001
	SEM = 0.744	SEM = 0.657	SEM = 0.663	SEM = 0.88	SEM = 0.146	SEM = 0.296	SEM = 0.33	

Note: BG—Polish White-Backed; RP—Polish Red; ZB—Polish Black and White; ZR—Polish Red and White; HO—Holstein-Friesian; SM—Simmental; JE—Jersey; FL—functional longevity; L1—share of the yield in the first 305-day lactation in lifetime yield; L2—share of the yield in the first complete lactation in lifetime yield; IL—longevity index (ratio of days in milk to length of life). Different lowercase letters in the same column indicate statistical differences between breeds (*p* < 0.05). SEM—standard error of mean.

**Table 13:** Breed differences in correlations for the number of days of productive life and life with the lifetime yield of milk and its chemical composition in breeds.

	BG	RP	ZB	ZR	HO	SM	JE
Productive life (days)							
Milk yield (%)	0.78 *	0.88 *	0.91 *	0.88 *	0.82 *	0.89 *	0.94 *
Fat yield (kg)	0.79 *	0.86 *	0.90 *	0.86 *	0.86 *	0.89 *	0.96 *
Fat content (%)	−0.02	0.08	0.02	0.08	0.13 *	−0.03	0.04
Protein yield (kg)	0.78 *	0.89 *	0.90	0.89 *	0.83 *	0.88 *	0.95 *
Protein content (%)	0.00	0.17 *	0.01	0.17 *	−0.01	−0.14 *	0.00
Lactose yield (kg)	0.75 *	0.86 *	0.90 *	0.86 *	0.81 *	0.87 *	0.93 *
Lactose content (%)	−0.18 *	0.15	−0.28 *	0.15	−0.20 *	−0.19 *	−0.12 *
Dry matter yield (kg)	0.76 *	0.87 *	0.91 *	0.87 *	0.84 *	0.88 *	0.95 *
Dry matter content (%)	−0.05	0.15	−0.02	0.15	0.05 *	−0.12 *	−0.02
Length of life (days)							
Milk yield (%)	0.75 *	0.87 *	0.88 *	0.87 *	0.79 *	0.87 *	0.93 *
Fat yield (kg)	0.76 *	0.84	0.87 *	0.84 *	0.83 *	0.87 *	0.95 *
Fat content (%)	−0.02	0.07	0.01	0.07	0.15 *	−0.03	0.05
Protein yield (kg)	0.75 *	0.87 *	0.87	0.87 *	0.79 *	0.86 *	0.94 *
Protein content (%)	−0.02	0.18 *	−0.01	0.18 *	−0.01	−0.12 *	0.00
Lactose yield (kg)	0.72 *	0.85 *	0.87 *	0.85 *	0.78 *	0.85 *	0.92 *
Lactose content (%)	−0.19 *	0.15	−0.29 *	0.15	−0.22 *	−0.20 *	−0.14 *
Dry matter yield (kg)	0.73 *	0.85	0.88 *	0.85 *	0.80 *	0.86 *	0.94 *
Dry matter content (%)	−0.06	0.15	−0.03	0.15	0.06 *	−0.11 *	−0.02

Note: BG—Polish White-Backed; RP—Polish Red; ZB—Polish Black and White; ZR—Polish Red and White; HO—Holstein-Friesian; SM—Simmental; JE—Jersey; * statistically significant (*p* < 0.05).

**Table 14:** Breed differences in correlations between milk yield and chemical composition in the first complete lactation and the number of days of productive life and of life.

	BG	RP	ZB	ZR	HO	SM	JE
Productive life (days)							
Milk yield (%)	−0.17 *	−0.04	−0.19 *	−0.21 *	−0.16 *	−0.18 *	−0.10 *
Fat yield (kg)	−0.15 *	−0.08	−0.19 *	−0.24 *	−0.12 *	−0.19 *	−0.10 *
Fat content (%)	0.02	0.03	−0.06	−0.10	0.11 *	−0.05	0.01
Protein yield (kg)	−0.18 *	−0.04	−0.18 *	−0.24 *	−0.16 *	−0.21 *	−0.11 *
Protein content (%)	−0.15 *	0.15	−0.03	−0.20 *	−0.09 *	−0.27 *	−0.08 *
Lactose yield (kg)	−0.23 *	−0.28 *	−0.19 *	−0.37 *	−0.18 *	−0.24 *	−0.13 *
Lactose content (%)	−0.28 *	−0.33 *	−0.08	−0.40 *	−0.18 *	−0.30 *	−0.20 *
Dry matter yield (kg)	−0.23 *	−0.30 *	−0.18 *	−0.39 *	−0.18 *	−0.25 *	−0.15 *
Dry matter content (%)	−0.26 *	−0.35 *	−0.03	−0.43 *	−0.13 *	−0.33 *	−0.25 *
Length of life (days)							
Milk yield (%)	−0.16 *	−0.03	−0.23 *	−0.21 *	−0.17 *	−0.16 *	−0.10 *
Fat yield (kg)	−0.14 *	−0.08	−0.22 *	−0.24 *	−0.13 *	−0.16 *	−0.09 *
Fat content (%)	0.01	0.02	−0.05	−0.12	0.13 *	−0.04	0.02
Protein yield (kg)	−0.18 *	−0.04	−0.22 *	−0.24 *	−0.18	−0.18 *	−0.11 *
Protein content (%)	−0.17 *	0.16 *	−0.03	−0.20 *	−0.09 *	−0.25 *	−0.07 *
Lactose yield (kg)	−0.23 *	−0.29 *	−0.23 *	−0.37 *	−0.20 *	−0.21 *	−0.13 *
Lactose content (%)	−0.28 *	−0.34 *	−0.10	−0.40 *	−0.19 *	−0.28 *	−0.20 *
Dry matter yield (kg)	−0.22 *	−0.30 *	−0.22 *	−0.39 *	−0.19 *	−0.22 *	−0.15 *
Dry matter content (%)	−0.27 *	−0.35 *	−0.03	−0.42 *	−0.11 *	−0.31 *	−0.25 *

Note: BG—Polish White-Backed; RP—Polish Red; ZB—Polish Black and White; ZR—Polish Red and White; HO—Holstein-Friesian; SM—Simmental; JE—Jersey; * statistically significant (*p* < 0.05).

## Data Availability

The datasets used and/or analysed during the current study are available from the corresponding author upon reasonable request.

## Abbreviations

The following abbreviations are used in this manuscript:

BG	Polish White-Backed
RP	Polish Red
ZB	Polish Black and White
ZR	Polish Red and White
HO	Polish Holstein-Friesian
SM	Simmental
JE	Jersey
LI	Longevity index
ML	Number of days in milk in the cow’s life
LT	Length of cow’s life (days)
L1	Percentage share of the yield in the first 305-day lactation in lifetime yield
MY305	Milk yield in the first 305-day lactation (kg)
LY	Lifetime milk yield (kg)
L2	Percentage share of the yield in the first complete lactation in lifetime yield
1FL	Yield in first complete lactation (kg)
L1D	Length of lactation (days)
